# Common models and approaches for the clinical educator to plan effective feedback encounters

**DOI:** 10.3352/jeehp.2022.19.35

**Published:** 2022-12-19

**Authors:** Cesar Orsini, Veena Rodrigues, Jorge Tricio, Margarita Rosel

**Affiliations:** 1 Norwich Medical School, Faculty of Medicine and Health Sciences, University of East Anglia, Norwich, UK; 2 Faculty of Dentistry, Universidad de Los Andes, Santiago, Chile; 3 Private Dental Practice, Santiago, Chile; Hallym University, Korea

**Keywords:** Feedback, Formative feedback, Medical education, Medical student

## Abstract

Giving constructive feedback is crucial for learners to bridge the gap between their current performance and the desired standards of competence. Giving effective feedback is a skill that can be learned, practiced, and improved. Therefore, our aim was to explore models in clinical settings and assess their transferability to different clinical feedback encounters. We identified the 6 most common and accepted feedback models, including the Feedback Sandwich, the Pendleton Rules, the One-Minute Preceptor, the SET-GO model, the R2C2 (Rapport/Reaction/Content/Coach), and the ALOBA (Agenda Led Outcome-based Analysis) model. We present a handy resource describing their structure, strengths and weaknesses, requirements for educators and learners, and suitable feedback encounters for use for each model. These feedback models represent practical frameworks for educators to adopt but also to adapt to their preferred style, combining and modifying them if necessary to suit their needs and context.

## Introduction

### Background/rationale

How should we approach feedback encounters as clinical educators? Which models or techniques could we use to give constructive and effective feedback to our learners and trainees? What should be the rationale behind the feedback approach? Undoubtedly, giving (and receiving) constructive feedback is crucial for learners to bridge the gap between their current performance and the desired standards of competence [1]. Ende [2] defines feedback in clinical education “as information describing students’ or house officers’ performance in a given activity that is intended to guide their future performance in that same or a related activity.” A number of authors have provided detailed principles and tips for giving constructive feedback in the clinical environment, emphasizing that feedback should be specific and goal-oriented; descriptive; non-judgmental; based on observed behaviors; provided in a sensitive, timely, and constant manner; manageable; actionable; and established as a dialogue [2,3].

It has been well established by several studies that constructive feedback drives learning and development [4], helps to gauge performance and make action plans for improvement [5], supports competence and autonomous motivation [6], and reconstructs knowledge and enhances clinical performance [7]. On the contrary, when non-constructive or no feedback is given, good practice is not reinforced, and performance might deteriorate [3], and learners may adopt a feedback-avoidance stance in the absence of good educator-learner rapport [4]. Therefore, giving constructive and effective feedback is an essential skill that should be included in our educator toolbox. However, clinical educators in faculty development courses frequently cite feedback skills as the most significant challenge and as an area for improvement in their practice [8]. This is mainly due to limited knowledge and practice in using different feedback models/techniques, how to approach feedback encounters, and a reluctance to cause offence or provoke defensiveness [2].

### Objectives

Giving constructive feedback is a skill, and like any other skill, it can be learned, practiced, and improved. Therefore, this study aimed to explore 6 of the most common and accepted feedback models in clinical settings and assess their transferability to different clinical feedback encounters so that clinical educators can make an informed decision on how and when to use them. These are the Feedback Sandwich, the Pendleton Rules, the One-Minute Preceptor, the SET-GO model, the R2C2 (Rapport/Reaction/Content/Coach), and the ALOBA (Agenda Led Outcome-based Analysis) model. These models were selected by reviewing the literature on feedback models in clinical education and on the authors’ experience in delivering multiple faculty development workshops on the subject. We present a handy resource in Table 1 describing their structure, strengths and weaknesses, requirements for educators and learners, and suitable feedback encounters for use, for each model. These feedback models represent practical frameworks for educators to adopt but also to adapt to their preferred style, combining and modifying them, if necessary, to suit their needs and context.

### Ethics statement

This was not a study with human subjects; therefore, neither approval by the institutional review board nor obtainment of the informed consent was required.

## Six common feedback models: how and when?

Within the teaching and learning process, it is helpful for the clinical educator to explore several feedback models and techniques described in the literature for their applicability in clinical settings and analyze the transferability to their educational practice in macro- or micro-feedback encounters. Micro-feedback, also known as informal or unplanned encounters, corresponds to brief doses of feedback, between 1 and 5 minutes, usually following the daily performance of skills [9]. Macro-feedback, also known as formal or planned encounters, corresponds to less frequent but more detailed and structured feedback, between 5 and 20 minutes, and commonly occurs at the middle and end of a rotation or placement, or after a significant event such as a workplace-based assessment or a medical error [9]. Some of the most common and accepted feedback models are the Feedback Sandwich [10], the Pendleton Rules [11], the One-Minute Preceptor [12], the SET-GO model [13], the R2C2 [14], and the ALOBA model [15]. Other techniques have been developed; however, these are all based on and correspond to adaptations of the 6 models mentioned above [4]. Table 1 describes the 6 feedback models, from the most educator- to learner-centered, outlining their structure, strengths and weaknesses, the required educator expertise level, the learner reflection and self-assessment skills required, and the type of feedback encounter where they would be suitable to use.

The 6 models have similarities and differences in their structure and objectives for the feedback encounter, from the simplest and most educator-centered, such as the Feedback Sandwich, to the most complex and learner-centered models, such as the ALOBA. Several aspects must be considered as part of the decision-making process when choosing the ideal model for a feedback encounter.

### Feedback Sandwich

The Feedback Sandwich receives its name due to the 2 doses of positive/reinforcement feedback with 1 dose of critical/corrective feedback sandwiched between to make it more palatable and acceptable. It is a brief and highly structured model that requires low levels of feedback-giving expertise by the educator and low reflection and self-assessment skills by the learner, making it suitable for inexperienced educators and applicable in various feedback encounters. Its weaknesses lie in that it is educator-centered and a one-way transmission of information with no input from the learner [3,10].

### Pendleton Rules

Pendleton Rules are a modification of the Feedback Sandwich [3,11], where the educator’s comments are preceded by the learner’s reflections on what was good about their performance, and what were the areas for improvement. This model represents a structured and rigid dialogue that is less educator-centred than the Feedback Sandwich, appropriate to initiate learners on reflective practice and self-assessment skills, and suitable for educators with low feedback-giving expertise. Its limitations are linked to the inflexibility of the conversation and the anticipation of critical feedback. Though it is applicable in various situations, it is mainly recommended for macro-feedback encounters [3].

### One-Minute Preceptor model

One model particularly useful in micro-feedback encounters and busy clinical settings is the One-Minute Preceptor model [12], also known as the 5-step “micro-skills” model. It provides a brief and straightforward framework for teaching and giving feedback during patient care. The educator first receives a commitment from the learner on one specific aspect, such as the diagnosis or treatment plan, then probes for supporting evidence exploring the learners’ rationale, teaching general rules if necessary, and finally establishes a brief discussion reinforcing the positive aspects and correcting mistakes. This just-in-time feedback model facilitates the development of clinical reasoning and decision-making skills, preferably individually, requiring medium feedback-giving expertise from the educator to explore a single aspect and provide balanced feedback, and medium learner reflection and self-assessment skills.

### SET-GO model

The SET-GO model aids memoir for the sequence and is beneficial when giving feedback in group encounters [13]. It is based on descriptive and non-judgmental feedback, where the educator asks the observed learner and group to describe what they saw, further explores and contributes to these observations, and then refers back to the learner for possible solutions and reflections. The group then establishes the goals to achieve and offers suggestions on how to accomplish those objectives, which might include developing skills or rehearsing [4]. This model encourages peer feedback, establishes a dialogue, and facilitates vicarious learning through the experience of others. The downsides are that it requires enough time for everybody to contribute, learners themselves need to develop feedback skills, and the educator requires medium to high expertise to provide feedback and manage the group dynamics.

### R2C2 model

The R2C2 model has been specifically developed to give assessment- and performance-based feedback rather than based on daily practice or specific rotation moments [14]. The model establishes a dialogue by exploring an assessment result, its value, and the learners’ perception/reaction. The educator first builds rapport with the learner, creating a respectful and trustful climate, exploring the learners’ reactions to the assessment, and stimulating reflection and self-assessment. Subsequently, the educator explores the learners’ understanding of the contents and results of the assessment, and adopts a coaching stance, agreeing on solutions and an action plan. The R2C2 model provides a learner-centered framework that facilitates the acceptance of the assessment and the feedback received, requiring learners to look beyond the assessment result and therefore requiring medium to high reflection and self-assessment skills. The educator needs high feedback-giving skills, as he or she must be prepared to face negative reactions and fully understand the assessment’s purpose and content to be reviewed.

### ALOBA model

Finally, the ALOBA model aims to establish a learner-centered conversation or interview-type feedback guided by the learners’ agenda and learning needs complemented by the educators’ view [15]. The learner is first asked to reflect and identify his or her needs and agenda for the feedback encounter. The educator then encourages self-assessment and problem-solving skills, reinforces theory-practice links, and provides balanced feedback. A discussion of suggestions and alternatives to accomplish the learner’s objective and learning needs follows, and finally, the educator checks the learner’s acceptance, summarizes the encounter, and agrees on an action plan [4]. The ALOBA model is considered an evolution of the Pendleton Rules as it adds learner-centeredness and flexibility to the feedback encounter, where the learner is an active participant throughout rather than a passive recipient of suggestions. The learner requires high insight, reflection, and self-assessment skills to lead the discussion and identify his or her needs and agenda. The educator requires high feedback-giving skills and judgement to facilitate the conversation and provide balanced feedback and theory-practice links.

These feedback models, with their strengths and weaknesses, represent practical frameworks for clinical educators to adopt but also to adapt to their preferred style. The models may be combined and modified to suit educators’ and their learners’ needs, considering the context in which feedback is given, the educator’s expertise, and the learner’s insight, reflection, and self-assessment skills. However, irrespective of the model used, clinical educators should always consider the aspects listed below when giving feedback [2,3,16].

**Common features to consider for an effective feedback encounter**

(1) Establish a safe feedback environment encounter.

(2) Base feedback on direct observation and provide it in a timely manner.

(3) Establish learners’ needs, goals, and self-assessment, and the objective of the feedback encounter.

(4) Provide balanced feedback (positive/critical aspects) as a dialogue, including descriptive information on what and how learners are doing (or not doing) in their efforts to reach a goal.

(5) Establish theory-practice links, recognizing “teachable moments.”

(6) Check learners’ understanding and acceptance of the feedback.

(7) Agree on an action plan.

(8) Document the encounter and plan a follow-up/subsequent feedback encounter.

## Conclusion

Giving feedback is critical for learners’ development, and educators play a crucial role in planning and providing constructive feedback encounters. Clinical educators should consider these feedback models, practices, and incorporate them into practice, reflecting on their performance and seeking feedback on their feedback skills from learners, peers, and/or trusted colleagues.

## Figures and Tables

**Table 1.** Feedback models, detailing their structure, strengths, weaknesses, educator and learners’ requirements, and suitable feedback encounters in which to be used

Model	Structure	Strengths	Weaknesses	Educator feedback-giving expertise	Learner reflection and self-assessment skills	Useful in which type of feedback encounters?
Feedback Sandwich	1. Educator provides a dose of positive/reinforcement feedback.	- Acceptable by learner as the impact of critical feedback is cushioned by the positive feedback	- Anticipation and increased tension knowing that critical feedback will be received.	Low	Low	- Micro- or macro feedback
	2. Educator provides a dose of critical/corrective feedback.	- Highly structured and easy to apply when time is limited and during clinical activities.	- Mostly focused on the educator, more monologue than dialogue.			- Written or verbal, individual or group
	3. Educator provides a dose of positive/reinforcement feedback	- Useful with passive/low-insight learners and for inexperienced educators.	- False-positive if encounter is mostly focused on reinforcement/positive feedback.			
Pendleton Rules	1. Educator asks learner what was good in his or her performance.	- Safe environment created by covering positive aspects first and then those that should be improved, from the perspective of the learner and educator.	- Anticipation and increased tension knowing that critical feedback will be received.	Low	Low	- Preferably macro- over micro-feedback
	2. Educator states areas of agreement and elaborates on good performance.	- A dialogue is established, although highly structured.	- Unsuitable in practice, during clinical care, but recommended in formal feedback encounters.			- Verbal
	3. Educator asks learner what was poor or could have been improved.	- Supports learners to initiate reflective practice and improve self-assessment skills.	- Risk of not covering aspects to improve when time is limited.			- Individual or group.
	4. Educator states what he or she thinks could have been improved.	- Useful with passive/low-insight learners and for inexperienced educators.	- The rigid structure prevents an interactive discussion and limits exploring or expanding on topics that might be relevant to the learner, risking becoming a passive recipient of suggestions, skills to develop, and action plans.			
One Minute Preceptor	1. Educator receives a commitment from learner (e.g., differential diagnosis, treatment plan).	- Effective use in practice, suitable for busy or time-constrained clinical environments.	- Variable duration of feedback encounter according to the needs of the learner and complexity of clinical case/scenario.	Medium	Medium	- Micro-feedback, verbal
	2. Educator probes for supporting evidence and explores learner’s rationale.	- Facilitates the development of clinical reasoning and decision-making skills.	- Does not allow exploration with a great level of detail or to expand on the learner’s agenda.			- Preferably individual over group feedback
	3. Educator teaches general rules.	- In a few minutes, it allows the educator to explore an aspect, reinforce knowledge/skills and provide balanced feedback.	- Unsuitable for formal feedback encounters.			
	4. Discussion with learner reinforcing what was done well.	- Just-in-time feedback.				
	5. Discussion with learner correcting mistakes.					
SET-GO	1. “What did I see?” Educator asks the observed learner and group to describe the situation/scenario/performance.	- Focuses on descriptive feedback to encourage a non-judgmental approach.	- Not recommended for individual feedback, though some of its elements could be transferred.	Medium to high	Medium	- Macro-feedback
	2. “What else did you see?” Further contributions are encouraged from the group and/or by the educator.	- Effective when delivering group feedback.	- Requires having enough time to involve the whole group.			- Verbal
	3. “What do you think?” Educator encourages learner to self-assess/problem-solve.	- Encourages peer feedback and joint problem-solving.	- Requires supervisor group facilitation skills.			- Group feedback
	4. “What goals are we trying to achieve?” Group discussion on outcome/objective.	- Focuses on the learner establishing a dialogue with the supervisor and peers.	- Unsuitable for informal feedback encounters.			
	5. “Offers on how to achieve goals.” Educator encourages group to discuss suggestions to achieve the goal.	- Facilitates vicarious learning and reflection through the experiences of others.	- Requires learners to develop feedback skills as the whole group is involved.			
R2C2	1. Educator builds a respectful and trustful relationship and establishes rapport with the learner.	- Effective when providing assessment- and performance-based feedback and reporting assessments.	- Unsuitable for informal feedback encounters.	High	Medium to high	- Macro-feedback
	2. Educator explores the learner’s reactions to the assessment/performance report, stimulating self-assessment and reflection.	- Empowers learners, stimulates reflection, facilitates acceptance of assessment results and the use of the feedback.	- Requires learners’ insight “to look” beyond the assessment results.			- Verbal.
	3. Educator explores the learner’s understanding of the contents of the assessment/performance report and results.	- A dialogue is established by exploring the assessment results, its value, and learner’s perception/reactions.	- Requires a skilled educator to be non-judgmental when exploring the content and learner’s reactions to the assessment results.			- Preferably individual over group feedback
	4. Educator adopts a coaching stance to agree on solutions and action plans.	- Provides a framework for feedback in defensive-stance situations.	- Enough protected time needed to explore the learner’s context/situation, and to establish rapport and a safe environment.			
		- A joint educator-learner action plan is developed in response to the assessment results.	- Educator must be prepared for negative reactions and must fully understand the purpose and content of the assessment/performance to be reviewed.			
ALOBA	1. Learner is asked to reflect on and identify his or her learning needs, objectives, and agenda for the feedback encounter.	- Priority is given to the learner’s objectives and agenda, complemented by the educator’s vision and agenda.	- Unsuitable for informal feedback encounters, enough protected time needed.	High	High	- Macro-feedback
	2. Educator encourages learner to self-assess, reflect on their situation, and problem-solve.	- Supports learners’ self-assessment, reflection, and clinical reasoning skills.	- Shares the disadvantages of the SET-GO model when it is used for group feedback.			- Verbal.
	3. Educator reinforces theory-practice links and delivers descriptive and balanced feedback.	- Established as a dialogue and interview style, where the learner is active in the skills and action plans to follow.	- More suitable for individual than group feedback encounters.			- Preferably individual over group feedback.
	4. Educator and learner discuss suggestions and alternatives to reach the objective and learning needs.	- Theory-practice links are discussed.	- Requires developed insight and reflective skills in learners so they may identify their agenda and learning needs.			
	5. Educator checks feedback acceptance, provides a summary and they agree on the action plan.	- Focused on the learner and their needs, creating a safe environment.	- Educator requires advanced disciplinary knowledge/skills to provide theory-practice links.			
		- A joint educator-learner action plan is developed focused on the learner’s objectives and needs.	- Developed skills and judgement by the educator to provide balanced feedback.			

R2C2, Rapport/Reaction/Content/Coach; ALOBA, Agenda Led Outcome-based Analysis.

## Supplementary materials

Supplementary files are available from Harvard Dataverse: https://doi.org/10.7910/DVN/APCRCN

Supplement 1. Audio recording of the abstract.
